# Endoscopic full-thickness resection of a gastrointestinal stromal tumor using a double-endoscope snare technique

**DOI:** 10.1055/a-2722-0953

**Published:** 2025-10-30

**Authors:** Jade Wang, Jeong Hoon Kim, Kamal Hassan, Kartik Sampath

**Affiliations:** 1 12295 Gastroenterology and Hepatology, Weill Cornell Medicine, New York, United States

## Introduction

Endoscopic full-thickness resection of a gastrointestinal stromal tumor using a double-endoscope snare techniqueVideo 1

Endoscopic submucosal dissection (ESD) with endoscopic full-thickness resection (EFTR) is an alternative to surgery for removing gastrointestinal stromal tumors (GISTs) 1 . We describe a case of successful EFTR using a novel double-endoscope snare traction technique ( Video 1 ).

## Case report

**Fig. 1:**
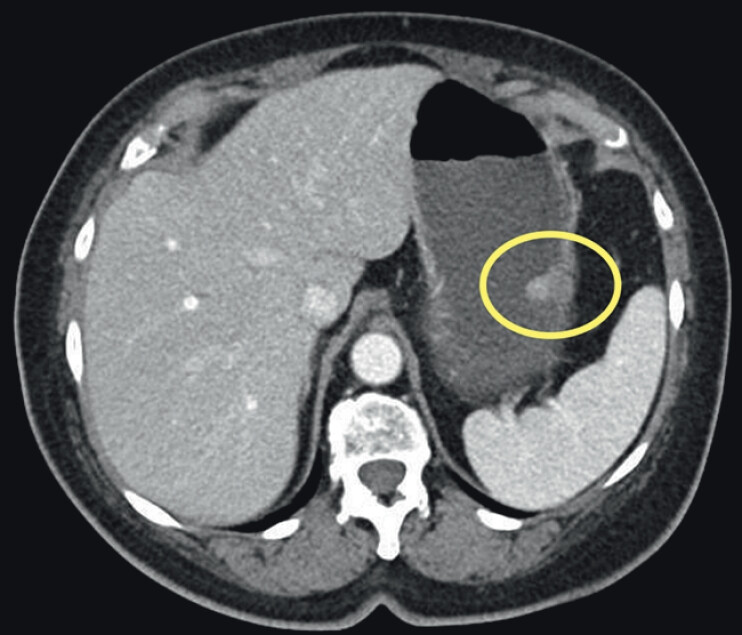
Computed tomography of the abdomen/pelvis revealing a 2.5-cm gastrointestinal stromal tumor (GIST) localized to layers 3 and 4.

**Fig. 2:**
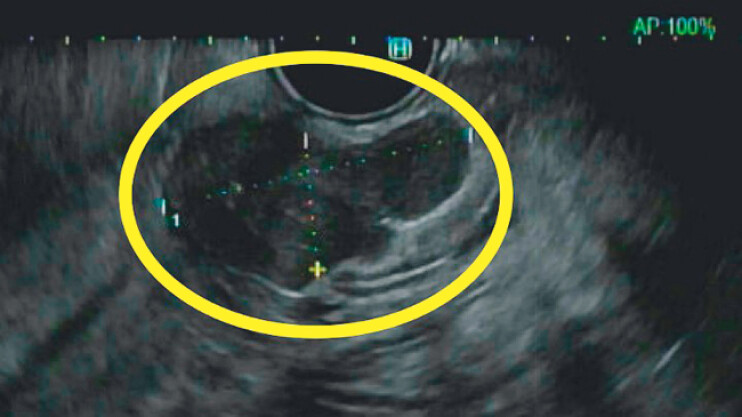
Endoscopic ultrasound (EUS) showing a 2.5-cm GIST.

A 76-year-old woman presented with abdominal pain. Computed tomography of the abdomen/pelvis ( Fig. 1 ) and esophagogastroduodenoscopy/endoscopic ultrasound (EGD/EUS) ( Fig. 2 ) with fine needle biopsy revealed a 2.5-cm GIST. A plan was made to proceed with ESD in an endoscopy suite with general anesthesia in supine position. The mucosa was dissected along the caudal edge, revealing an exophytic lesion from the muscularis propria. EFTR was performed to remove the lesion en bloc. One edge of the lesion remained tethered proximally. A snare was inserted to grab the lesion and provide traction to fully expose the lesion. The scope was removed per os. A second gastroscope was inserted alongside the snare holding traction and the proximal end of the lesion was dissected completely via IT2 knife. The scope was removed per os with the snare attached to the lesion, preventing migration into the peritoneum. The defect was closed via two-layer endoscopic suturing, allowing durable full-thickness closure and avoiding clipping eversion, a potential consequence of the alternative clip-loop technique.

Post-closure gastrogram was negative for leak. A subsequent upper gastrointestinal series was negative for gastric outlet obstruction. Pathology revealed tumor at the cauterized resection margin, confirmed by the pathologist to be expected, given the resection technique and consistent with en-bloc removal with preserved capsule. At 12-day follow up, the patient was recovering well.

## Conclusions

This case supports the efficacy of the double-endoscope snare traction technique 2 for removal of well-circumscribed GISTs. It allows the endoscopist to more easily manipulate the tumor, prevents tissue loss into the peritoneum, and facilitates visualization of the dissection plane. Two-layer endoscopic suturing can effectively provide durable defect closure without leak.
